# A case presentation of duodenal gastric epithelial neoplasm–fundic-gland mucosa lesions

**DOI:** 10.1055/a-2724-6813

**Published:** 2025-11-19

**Authors:** Chenchen Zhang, Zhaosheng Chen, Nan Zhang, Lulu Zhang, Honglei Wu

**Affiliations:** 1 66555 Department of Gastroenterology, The Second Qilu Hospital, The Second Clinical Medical School of Shandong University, Jinan, China; 2 66555 Department of Gastrointestinal Endoscopy Center, The Second Qilu Hospital, The Second Clinical Medical School of Shandong University, Jinan, China; 3 66555 Department of Pathology, The Second Qilu Hospital, The Second Clinical Medical School of Shandong University, Jinan, China

A 63-year-old man was referred to our institution with reports of a superficial nonampullary duodenal epithelial tumor (SNADET). Detailed gastroscopy 1 revealed a 10 mm, reddish, slightly elevated margin with a central shallow depression lesion (Paris 0–IIa + IIc) in the duodenum Fig. 1 **a, b** ). Under magnifying endoscopy with narrow-band imaging (NBI), irregular microsurfaces and microvascular patterns suggested it as early cancer ( Fig. 1 **c–e** ). A contrast-enhanced CT scan revealed no signs of metastasis or any abnormalities. Endoscopic submucosal dissection (ESD) was performed by an experienced endoscopist using a jet-functional gastroscope (GIF-Q260J; Olympus Medical Corporation, Tokyo, Japan). En bloc resection of the lesion was performed ( Video 1 , Fig. 1 **f** ).

**Fig. 1:**
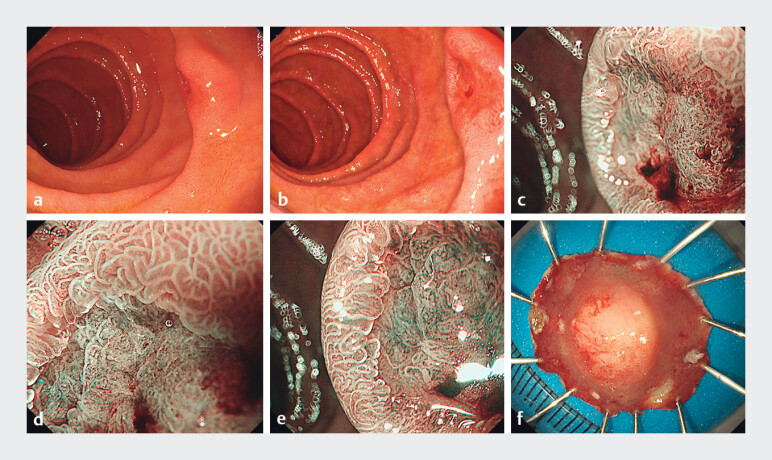
Endoscopic features of the lesion. A and B, White light view: **a** 10-mm, well-demarcated, reddish, slightly elevated margin with a central shallow depression lesion (Paris 0–IIa + IIc) in the descending duodenum. **c, d** and **e** , Magnifying endoscopy findings with narrow-band imaging (NBI): the demarcation line was present, and the surrounding mucosa of the tumor was regular villi with a light-blue crest. The marginal crypt epithelium (MCE) of the lesion was non-uniform in shape and arranged irregularly. The MCE is vague in the intervening part (the protrusion between crypts). The vessels in the lesion are non-uniform in shape, dilated and circuitous, distributed asymmetrically, and arranged irregularly. According to the vessel plus surface (VS) classification system, the lesion was diagnosed as cancer. **f** Pinned intact resection specimen, macroscopically showing en bloc resection.

Gastroscopy showing endoscopic features of GA-FGM and the procedure of endoscopic submucosal dissection (ESD). LBC, light blue crest; MCE, marginal crypt epithelium; VEC, vessels within an epithelial circle.Video 1

Histopathologic findings of the postoperative specimen were as follows: The tumor exhibited a lobulated growth pattern, herniating into the muscularis mucosae, with glands demonstrating significant architectural atypia characterized by irregular branching and labyrinthine configurations. The majority of cells displayed gastric fundic gland-type differentiation, while focal surface cells exhibited gastric foveolar-like differentiation. Tumor cells were exposed on the surface ( Fig. 2 ). Immunohistochemical staining revealed that MUC6 was diffusely expressed in the tumor area, while MUC5AC was expressed in the surface and deeper layers. Pepsinogen I and H ^+^ K ^+^ -ATPase ^+^ were scattered throughout the tumor area. And MUC2 was negative. ( Fig. 3 ). The above findings indicated differentiation into both the gastric fundic gland and gastric foveolar epithelium, confirming the diagnosis of gastric adenocarcinoma of fundic gland mucosa (GA-FGM) type 2 3 . Curative endoscopic resection was performed using ESD in the absence of lymphovascular invasion and negative resection margins.

**Fig. 2:**
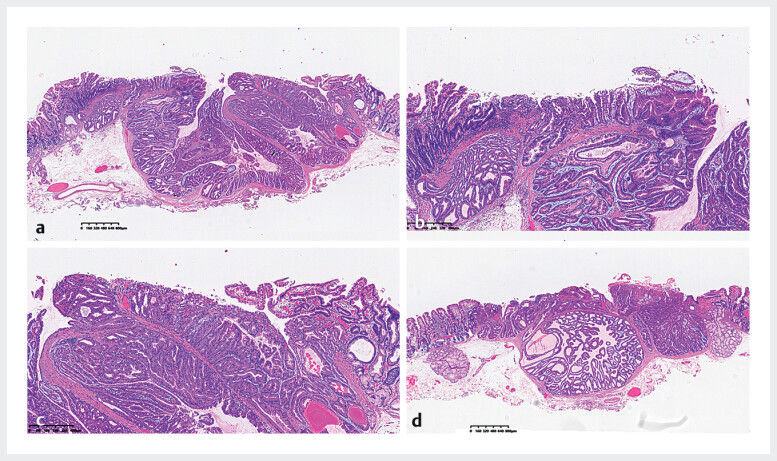
Histopathological findings of endoscopically dissected specimen in H&E staining. The tissue construct of the foveolar epithelium and fundic gland was collapsed; the mucosal layer architecture was destroyed; daedaleous tumor glands with irregular branching and dilatation were observed in the superficial mucosal layer. **a, d** : 4× magnification; **b, c** 10× magnification.

**Fig. 3:**
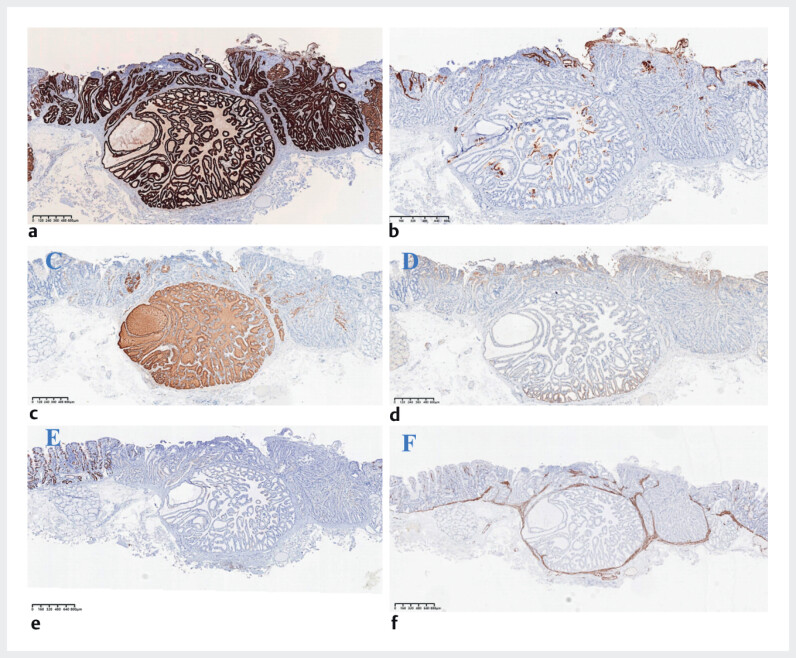
Immunohistochemical staining findings. **a** The tumor shows strong positivity for MUC6, **b** partial positivity for MUC5AC in the superficial and deeper layer, **c** scattered positivity for pepsinogen I, **d** and weak positivity for H ^+^ K ^+^ -ATPase ^+^ . **e** MUC2 is negative, while **f** Desmin staining showed that the tumor was located within the mucosa.

To the best of our knowledge, this is the first report of a GA-FGM in the duodenum. Both endoscopic and histopathological features of the lesion are presented, which have substantial clinical and pathological value.

Endoscopy_UCTN_Code_CCL_1AB_2AZ_3AB

## Notes

Endoscopy E-Videos https://eref.thieme.de/e-videos
*E-Videos* is an open access online section of the journal *Endoscopy* , reporting on interesting cases and new techniques in gastroenterological endoscopy. All papers include a high-quality video and are published with a Creative Commons CC-BY license. Endoscopy E-Videos qualify for HINARI discounts and waivers and eligibility is automatically checked during the submission process. We grant 100% waivers to articles whose corresponding authors are based in Group A countries and 50% waivers to those who are based in Group B countries as classified by Research4Life (see: https://www.research4life.org/access/eligibility/ ). This section has its own submission website at https://mc.manuscriptcentral.com/e-videos .
